# A feasibility study of 70 kV double low-dose coronary imaging technique in abdomen-fatty patients using dual-source CT

**DOI:** 10.4314/ahs.v23i3.70

**Published:** 2023-09

**Authors:** Lei Chen, Shang Ge, Yuan Chen, Ting-Ting Zhang, Zhao-Huan Zhu

**Affiliations:** Department of Radiology, The Affiliated Huaian No.1 People's Hospital of Nanjing Medical University, Huaian 223300, China

**Keywords:** Radiation dose, tomography, coronary artery, iterative reconstruction, contrast agent, abdominal obesity

## Abstract

**Objective:**

To investigate the aplication of low contrast agent concentration and low tube voltage in coronary CTA on patients with high BMI (26kg/m^2^<BMI≤28kg/m^2^) and ultra-high BMI (BMI>28kg/m^2^).

**Methods:**

60 patients with high BMI (26kg/m2<BMI≤28kg/m2) and 60 patients with ultra-high BMI (BMI>28kg/m^2^) were randomly divided into two groups: double low group A (n=30, tube voltage = 70 KV), double low group B (n=30, tube voltage = 70 KV), processed by SAFIRE iterative reconstruction with 270 mgI/ml contrast agent. Conventional group a (n=30, tube voltage = 120 KV), conventional group b (n=30, tube voltage = 120 KV), with filtered back projection (FBP) and 370 mg I/ml contrast agent. the image excellent index (FOM), the effective radiation dose (ED), mean CT value, signal-to-noise ratio (SNR), and contrast-to-noise ratio(CNR) between corresponding groups were compared.

**Result:**

There was no significant difference in subjective scores of coronary artery image quality between the two high BMI subgroups (P>0.05).The ED of group A (1.103±0.101 mSv) was significantly lower than that of group a (4.663±0.412 mSv) (P<0.001).There was no significant difference in mean CT value, SNR and CNR between the two subgroups (P>0.05).The image excellent index (FOM) of group A was higher than that of group a (P<0.05).The total iodine content and iodine injection rate in group A were lower than those in group a (P<0.001).The difference of subjective scores of coronary artery image quality between the two ultra-high subgroups was significant. The mean CT value, SNR and CNR of group B were lower than those of group b (P<0.05). The images of 14 patients in group B could not meet the diagnosis demand.

**Conclusion:**

It is feasible to reduce the tube voltage to 70KV in patients with abdominal BMI with high BMI (26Kg/m^2^<BMI≤28Kg/m^2^). For patients with abdominal obesity with ultra-high BMI (BMI>28Kg/m^2^), under the same conditions, the 70KV can not meet the daily diagnosis requirement.

## Introduction

Coronary computerized tomography angiography (CCTA) has been widely used in the diagnosis and screening of coronary artery disease with its non-invasive advantages 1-5. However, the higher radiation dose during the inspection process has not been neglected. Based on the principle of dose optimization (ALARA) 6 and the principle of constrained radiation dose proposed by the International Commission on Radiological Protection (ICRP) 7, it is necessary to reduce the radiation dose and the amount of contrast agent to maximize the protection of patients. Obese patients are common in this type of examination. However, these population have higher X-rays absorption rates, higher radiation doses and image noise, the image quality and diagnostic efficiency will decrease.

Decreasing radiation dose is a long-standing challenges. Double low-dose scanning protocol refers to “low tube voltage” and “low contrast agent dosage” combined with iterative reconstruction technology to obtain image quality that can meet the needs of clinical diagnosis. Thus it can reduce the radiation dose and iodine contrast agent dosage. Previous 8 double low-dose studies showed that for obese patients with high BMI (26kg/m^2^<BMI<30kg/m^2^), the tube voltage can be reduced from 120 KV to 80 KV, and the images obtained can still meet the subjective diagnosis needs. A large amount of fat in abdominal obesity patients accumulates in the abdomen, and the morphology of the chest is similar to that of normal BMI patients. When coronary CTA is performed on such population, can the tube voltage be further reduced to 70KV and remain diagnostic images? There have been only a few reports in the past 8.

Although the BMI range in the study was set to 26kg/m^2^<BMI<30kg/m^2^, the average BMI level was not very high, for most population were concentrated in 26kg/ m^2^<BMI≤28kg/m^2^. The study using isotonic low concentration contrast agent (270mg I / ml) and low tube voltage (70KV) joint iterative reconstruction technology (SAFIRE-4), perform CCTA examination on patients with abdominal obesity with high BMI (26Kg/m^2^<BMI≤28Kg/m^2^) and ultra-high BMI (BMI>28Kg/m^2^) to observe the feasibility of 70KV .

## Materials and methods

### Case data

Prospectively and continuously collected 120 cases of abdominal obesity patients with clinically suspected coronary artery disease from July 2016 to September 2018, waist circumference men>85cm, women>80cm 9. Sixty patients with high BMI (26kg/m^2^<BMI≤28kg/m^2^) and 60 patients with ultra-high BMI (BMI>28kg/m^2^) were randomly divided into two groups: double low-dose group A has 12 male and 18 female patients; double low-dose B group has 13 male and 17 female patients; routine group a has 16 male and 14 female subjects; conventional group b, 15 male and 15 female subjects. Exclusion criteria 8: patients with coronary artery disease; those who are allergic to iodine contrast agent; those with severe arrhythmia, cardiac dysfunction; those with liver and kidney dysfunction (serum creatinine >12mol/L); pregnant women; body mass index (BMI)≤26 kg / m^2^; cannot cooperate with the breather. All patients or family members who were examined signed an informed consent form prior to the examination. This study has been reviewed and approved by the ethics committee of Affiliated Huaian No.1 People's Hospital of Nanjing Medical University (Approved #: NJYY20160316008).

### Inspection method

**Preparation of coronary artery CTA before the scan:** Take sublingual nitroglycerin 0.5mg. The 18G indwelling needle was placed in the median vein of the right elbow, and the patients were placed in the supine position after breath-holding training .

**Scanning equipment and parameters:** Use German Somatom Definition Flash. All groups used adaptive forward-looking ECG-gated scanning sequence (CorAdSeq); The scanning range is 1 cm below the tracheal bifurcation to the diaphragmatic surface. The ECG phase is automatically selected based on the heart rate after holding breath. Group A and group B used contrast agent iodixanol (270 mg I/mL), contrast tagent dosage 1 mL/kg; group a and group b used contrast agent iopromide (370 mg I/mL), contrast agent dosage 1 mL/kg. The injection rate is 5 ml/s. The tracking trigger technique was used in both groups. The region of interest was placed at the root of the ascending aorta. The trigger threshold was 100 HU and the delayed scan time was 6s. The tube voltage of group A and group B is 70KV, and the tube voltage of group a and group b is 120KV; the tube current is automatically modulated by four-dimensional intelligent real-time dose regulation technology (CARE Dose 4D, Siemens Medical System). The detector is collimated 2mm×64mm, the image layer thickness is 0.75mm, the convolution kernel b26f. When the heart rate <75 beats/min, the phase is between 65% and 75% RR interval, and the acquisition phase is 40%-50% RR interval when the heart rate ≥75 beats/min. Group A,B use the Sinogram Affirmed Iterative Reconstruction (SAFIRE) algorithm, and Group a,b use the Filtered Back Projection (FBP) algorithm. d

### Image post processing

All images were processed using the Circulation software. Perform volume rendering, maximum density projection, multi-planar reconstruction, and surface reconstruction. The CT values of the aortic root, left main trunk, left anterior descending branch, left circumflex artery, and right coronary artery were measured in both groups.

### Image quality evaluation

**Objective evaluation indicators:** the region of interest (ROI) is placed at the proximal segment of each main blood vessels, and the ROI should be as large as possible avoiding calcification, plaque and wall. (1) Signal to noise ratio (SNR) = blood vessel CT value / vascular noise; (2) Contrast signal to noise ratio (CNR) = (vascular CT value - anterior chest wall muscle ct value) / vascular noise; (3) Image noise is the SD value of the region of interest of the ascending aorta;(4) Image excellent index 1 (FOM), FOM = CNR^2^ / effective dose (ED).

Subjective evaluation indicators refer to the American Heart Association (AHA) 15 segment of the coronary artery tree 10. All coronary segments whose diameter≥1.5 mm were selected for analysis. The image quality evaluation criteria are as follows: the edges of the image are clear, and no motion artifacts are 5 points: the edges of the image are slightly blurred, and the slight motion artifacts are 4 points; the edges of the image are moderately blurred, and there are moderate motion artifacts, but there is no obvious error. The layer does not affect the diagnosis of 3 points; the edge blur and motion artifacts are 2 points: the coronary lumen is unrecognizable which cannot be diagnosed is 1 point. Those with 3 or more points are diagnosable images.

**Radiation dose calculation:** The volume CT dose index (CTDI) and the dose-length product (DLP) are provided by the scanner dose report, and the effective radiation dose ED (mSv)=DLP×k is calculated, where k 11 represents the conversion coefficient, refer to the European guideline for a mean of 0.014 mSv/(mGy·cm). The use of size specific dose estimates (SSDE) is now advocated internationally to more accurately assess the radiation dose received by the subject 12.

**Iodine intake:** Calculation of iodine intake including total iodine and iodine injection rate, calculation formula 2: Total iodine amount (g) = contrast agent concentration (mg/ml) × contrast agent usage (ml) /1000; iodine injection rate (g/s) = contrast agent concentration (mg/ml) × contrast agent injection rate (ml/s)/1000.

### Statistical analysis

The results of the study were processed by SPSS 22.0 software. Measurement data ***X̅ ± s*** is expressed in absolute numbers ( ***X̅ ± s***). The general baseline data , the scanning parameters and the objective evaluation indexes were analysed by two independent samples t-test, and the subjective evaluation index was tested by rank sum test; the consistency of two doctors' assessment was evaluated using Kappa test. The difference was statistically significant at P < 0.05.

## Results

**Baseline data analysis:** There was no significant difference in age, heart rate, body mass index (BMI) and Z-axis scan between the two groups (P>0.05, Table 1).

**Table 1:** Comparison of general data of four groups of patients (*X̅ ± s*)

	Age	Heart rate	BMI	Z-axis range
Group A (n=30)	56.1±7.2	74.0±9.9	27.3±1.1	15.36±2.1
Group a (n=30)	55.3±11.1	75.2±8.0	27.9±1.2	15.94±2.4
*t value*	-0.513	-0.327	0.325	0.456
*p value*	0.126	0.219	0.366	0.361
Group B (n=30)	59.1±12.2	76.0±8.9	29.3±1.1	15.19±1.2
Group b (n=30)	59.3±12.1	75.2±9.0	29.9±1.7	15.33±1.6
*t value*	-0.383	-0.106	0.671	0.377
*p value*	0.305	0.403	0.103	0.132

**Radiation dose and iodine intake:** The comparison of radiation dose, iodine intake are listed in Table 2.

**Table 2:** Comparison of radiation doses in four groups of patients (*X±s*)

	Tube current (mA)	CTDlovl (mGy)	DLP (mGy cm)	ED (mSv)	EDssde (mSv)	Total iodine (g)	Iodine injection rate (g/s)
Group A (n=30)	397.6±30.5	4.39±1.13	58.23±10.13	0.803±0.201	1.103±0.101	15.38±2.10	1.42
Group a (n=30)	308.7±49.9	16.26±2.05	191.60±28.09	3.028±0.503	4.663±0.412	25.13±4.09	1.98

*t value*	9.236	-14.184	-15.320	-17.392	-11.791	-7.375	-
*p value*	<0.001	<0.001	<0.001	<0.001	<0.001	<0.001	-

Group B (n=30)	427.6±29.5	4.29±1.03	57.20±9.31	0.799±0.302	1.137±0.210	15.68±1.99	1.46
Group b (n=30)	363.2±43.1	16.77±2.20	198.67±33.20	3.197±0.632	4.863±0.537	25.97±2.98	2.01

*t value*	11.237	-15.996	-17.001	-19.360	-12.308	-7.958	-
*p value*	<0.001	<0.001	<0.001	<0.001	<0.001	<0.001	-

Note: CTDlov1 volume CT doze index; DLP is the dose length product; EDssde is the body-dependeant effective radiation dose

The CTDlovl, DLP, ED EDssde value, total iodine, and iodine injection rate were significantly lower in low KV groups.(P<0.001).

### Quality evaluation

**1. Objective evaluation:** The objective scores of the coronary segments in the four groups were compared in Table 3. The CT values of all patients in groups A, a and B,b were >300 HU. There was no significant difference in CT value, SNR and CNR (P>0.05). Compared with group a, the FOM of group A was significantly higher (t=11.364, 12.729, 11.663, 12798, P<0.05); the mean CT value, SNR, CNR and FOM of group B were lower than group b (P<0.05).

**Table 3:** Comparison of objective quality evaluation of images of four groups of cases (*X±s*)

Group	n	RCA				LM			

		CT value (hu)	SNR	CNR	FOM	CT value (hu)	SNR	CNR	FOM
Group A	30	390.6±40.3	13.99±4.37	11.99±2.81	208.9±20.1	381.1±36.2	12.83±2.61	11.65±3.20	219.7±15.3
Group a	30	397.5±42.5	14.68±4.10	12.32±3.10	82.2±19.8	388.0±42.3	13.03±2.92	12.00±2.82	83.3±16.4

*t value*		0.353	1.268	3.691	11.364	0.203	0.107	0.230	12.729
*p value*		>0.05	>0.05	>0.05	<0.05	>0.05	>0.05	>0.05	<0.05

Group B	30	220.5±63.3	8.31±3.31	7.21±3.01	64.9±19.63	219.1±62.3	8.10±3.49	7.65±3.80	69.3±18.03
Group b	30	384.5±42.5	14.70±5.32	12.11±2.96	77.3±18.7	382.7±46.4	14.90±3.11	13.01±2.06	82.31±19.01

*t value*		-7.604	-9.321	-8.696	-0.963	-8.069	-9.036	-8.663	-1.039
*p value*		<0.05	<0.05	<0.05	<0.05	<0.05	<0.05	<0.05	<0.05

Group	n	LAD				CX			

		CT value	SNR	CNR	FOM	CT value (hu)	SNR	CNR	FOM
Group A	30	380.9±42.7	13.29±6.12	12.87±3.91	213.0±18.0	372.3±32.1	14.69±5.10	13.10±1.86	238.0±11.1
Group a	30	385.2±47.1	13.36±5.35	12.12±4.61	86.3±13.6	384.1±41.8	15.03±4.41	14.71±2.10	88.9±13.7

*t value*		0.215	1.608	2.347	11.663	0.326	0.512	0.203	12.798
*p value*		>0.05	>0.05	>0.05	<0.05	>0.05	>0.05	>0.05	<0.05

Group B	30	241.9±109.7	8.10±4.70	7.77±5.91	71.3±17.9	199.3±82.1	8.99±4.30	7.15±1.96	68.0±14.1

Group b	30	403.2±49.1	12.26±5.71	11.96±4.61	79.2±13.1	399.0±39.8	12.51±4.92	14.98±2.30	81.9±12.9

*t value*		-7.258	-8.369	-6.314	-0.367	-8.912	-7.147	-8.921	-0.215
		
*p value*		<0.05	<0.05	<0.05	<0.05	<0.05	<0.05	<0.05	<0.05

Note: aa is ascending aorta; rca is right coronary artery; 1m is left main; lad is left anterior descending; cx is left circumflex; snr is signal to noise ratio; cnr is contrast signal to noise ratio.

**Figures 1:**
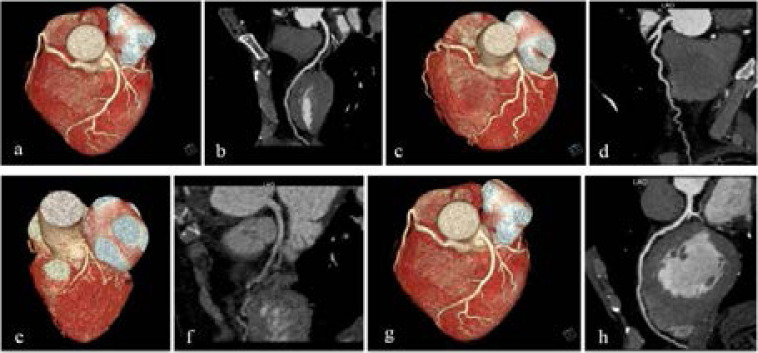
(a) & (b) are the same female patient from group A, 56 years old. (a): vr image, the coronary segment shows clear, no artifacts; (b): cpr image, the left front branch wall is slightly blurred, no artifacts, score 4 points (c) & (d) are the same female patient, 59 years old, from group a. (c): VR image, the coronary segment shows clear, no artifacts; (d): CPR image, the left anterior descending wall is smooth, no artifacts, score 5 points (e) & (f) are the same male patient, 62 years old from group B. (e): vr image, the proximal segment of the coronary segment is acceptable, the mid-distance segment is unclear, no motion artifacts; (f): cpr image, the left anterior descending lumen lumen density is low, and the surrounding tissue contrast is poor, although no Motion artifacts, but the diagnosis of plaque is difficult, scored 2 points (g) & (h) are the same male patient, 58 years old from group b. (g): VR image, the coronary segment shows clear, no artifacts; (h): CPR image, the right coronary artery wall is smooth, no artifacts, score 5 points

**2. Subjective evaluation:** The subjective image quality scores are listed in Table 4: The segments with anatomical variation or diameter <1.5 mm were not evaluated. Group A has 438 coronary artery segments. 97.7% (428/438) can be used for diagnosis. Group a has 440 coronary artery segments with 98.6% (434/440) can be used for diagnosis. In group B, 48.0% coronary artery segments (198/412) can be used for diagnosis, 98.4% (431/438) coronary segments in group b can be used for diagnosis. There was no significant difference in the subjective quality scores between the two high BMI groups (P>0.05). Significant difference exists in the subjective quality scores between the two ultra-high BMI groups (P<0.05). The coronary artery images of four subgroups are shown in Figures 1. The agreement between the two observers was good (Kappa=0.931, P>0.05).

**Table 4:** Comparison of subjective quality scores of four groups of images

	Number of image quality score segments (a)					

	5 points	4 points	3 points	2 points	1 point	Total
Group A (n=30)	212	166	50	6	4	438
Group a (n=30)	216	162	56	2	4	440

*f value*		0.315				
*p value*		>0.05				

Group B (n=30)	30	60	108	86	128	412
Group b (n=30)	210	155	66	4	3	438

*f value*		11.368				
*p value*		<0.05				

## Discussion

The obese patients is common in coronary CTA examination. These patients have higher image noise and lower signal-to-noise compared to conventional population 13. The image quality tends to be failed to meet the daily diagnostic needs. In order to improve the image quality, the tube voltage and the contrast agent dosage are often increased, which undoubtedly increases the radiation dose. On the other hand, high span style=“font-family: ‘Times New Roman’”>contrast dose also increases the risk of renal impairment 5,14,15, which does not meet the guiding principle of the dose optimization (ALARA) principle. Although the BMI value is high in abdominal obesity, the accumulation of fat is mainly concentrated in the abdomen, and the main action site of coronary CTA examination is the chest. The shape of the chest in this group is close to that of the normal BMI population, which provide a basis for reduction of radiation dose during coronary CTA examination.

A decrease in tube voltage will result in an exponential decrease in radiation dose 1,4,5,16. However, the reduction of tube voltage is bound to increase image noise. This contradiction is particularly evident in the examination of obese patients. The reduction of tube voltage while ensuring the image quality has been hot in recent years. Major companies have successively developed iterative reconstruction algorithms, such as the latest iterative model reconstruction (IMR) algorithm developed by Philips, and the SAFIRE iterative reconstruction algorithm from Siemens. In this study, SAFIRE iterative reconstruction technology was applied in image processing 17,18. It combines the iterative algorithm based on the original data and the image data. The algorithm compare image data with the ideal noise model, and then repeat denoising correction which could significantly reduce the image noise. Consistently, previous reports 1,2,19 showed that there are four kinds iterative levels to choose. The higher the intensity, the lower the image noise, but the “fuzzier” effect proceed image has. Therefore, it is recommended to use medium 20 strength iteration value (safire-4).

Previous studies showed 1,2 that the reduction of the tube voltage can effectively enhance the photoelectric and Compton scattering effect of the iodine-containing contrast agent, therefore lowering the tube voltage will increase the CT value of the contrast agent. However, the CT value of the blood vessel should not be as high as possible. It is generally considered that the vessel CT value >300 HU could suffice the daily diagnosis 20. This provides an idea for reducing the dosage of iodine contrast. This double low-dose scanning scheme, which simultaneously reduces the contrast agent concentration and lowers the tube voltage, is broadly and intensively investigated [8]. In the double low-dose studies, the obese patients CCTA examination can reduce the tube voltage from 120 KV to 80 KV, and remain imagine quality.

The dosage of iodine contrast agent could be greatly reduced thus decreasing the incidence of contrast agent nephropathy. However, for obese patients with high BMI (26kg/m2<BMI<30kg/m^2^), the feasibility of 70KV in the double low-dose scanning protocol is rare to be tested. The cases in this study were all abdominal obesity patients. The study refined the group according to the BMI range, using isotonic low concentration contrast agent (270mg I/ml) combined with low tube voltage (70KV). Iterative reconstruction technique (SAFIRE-4) was performed on patients with abdominal obesity with high BMI (26kg/m2<BMI≤28kg/m^2^) and ultra-high BMI (BMI>28kg/m2). The control group used 370mg I/ml contrast agent with conventional tube voltage (120KV), combined with FBP reconstruction technology. The two groups injected the contrast agent at the same flow rate. The results showed that the two physicians had good agreement (Kappa = 0.931, P < 0.05).

There was no significant difference in the subjective scores of coronary artery image quality between A and a group (P>0.05).The tube current of group A was significantly higher than that of group a, (P<0.001), the total radiation dose of group A (1.103±0.101 mSv) was still significantly lower than that of group a (4.663±0.412 mSv)(P < 0.001). There was no significant difference in mean CT value, SNR and CNR between the two groups (P>0.05). At present, in order to standardize and compare the effects of different radiation doses on image quality, the FOM concept is proposed 1. The image contrast, noise and radiation dose are considered together, and the higher the value, the higher the excellent index of the CT image. Compared with group a, the CNR value of coronary artery in group A did not decrease significantly, but due to the large decrease of ED value, the FOM value of the experimental group increased, and the image excellent index (FOM) of group A was higher than that of group a (P<0.05). The total iodine content and iodine injection rate in group A were lower than those in group a (P<0.001). It is suggested that the 70kV tube voltage can improve the excellent index of coronary CTA images in patients with abdominal obesity with high BMI (26kg/m2<BMI≤28kg/m^2^). There exists significant difference in the subjective scores of coronary artery image quality between the B and b groups (P<0.05).

In the B group, 14 patients had larger image noise, the CT value of the vascular cavity was smaller than that of the surrounding tissue, and the vessel wall was grainy. With strong sawtooth feeling ,the imagines were dissatisfied for missing small soft plaque. The mean CT value, SNR and CNR of group B were smaller than group b (P<0.05). Although the radiation dose and total iodine content of group B were lower than group b, the decreased subjective and objective evaluation suggested that 70KV is not suitable for ultra-high BMI population(BMI>28kg/m^2^) with abdominal obesity. This may be because the chest fat layer is thicker and the X-ray attenuation is stronger. Even with the iterative reconstruction technique, the image distortion is still high, which cannot meet the diagnostic requirements.

## Conclusion

In summary, for high BMI (26kg/m^2^ < BMI ≤ 28kg / m^2^) population with abdominal obesity, it is feasible to reduce the tube voltage to 70KV.It can remain imagine quality, reduce the radiation dose and iodine intake. For patients with ultra-high BMI (BMI>28kg/m^2^), 70KV will significantly reduce the quality of the image and dissatisfy daily diagnostic needs.

## Data Availability

Not applicable.
